# Supplementary material for: Modeling the dynamics of aeolian meter-scale bedforms induced by bed heterogeneities
Source: Proc Natl Acad Sci U S A. 2025 May 16;122(20):e2426143122. doi: 10.1073/pnas.2426143122 (PMC12107171; doi:10.1073/pnas.2426143122)
Supplement: Supplementary file 1 — Appendix 01 (PDF) [file pnas.2426143122.sapp.pdf]

PNAS

Supporting Information for

Modeling the dynamics of aeolian meter-scale bedforms induced by bed heterogeneities

Camille Rambert, Joanna M. Nield, Clément Narteau, Pauline Delorme, Giles F.S. Wiggs, Matthew C. Baddock, Jim Best, Ken T. Christensen and Philippe Claudin

Camille Rambert
E-mail: camille.rambert@espci.fr
Philippe Claudin
E-mail: philippe.claudin@espci.fr

This PDF file includes:

Supporting text
Figs. S1 to S18

Supporting Information Text

Appendix: Analytical expression for the flux profile over a flat sand patch

In the case of a perfectly flat patch subjected to a constant wind shear stress, the model can be solved analytically in order to obtain the longitudinal flux profile. $L_{\rm i}$ is the length of the erodible region, which starts at $x = 0$. Importantly, this idealized configuration is used for a better understanding of erosion and deposition processes associated with flux variations, not for the fit of bedform elevation profiles from the field data.

Long initial patch length. According to the linear form of the transition in transport capacity proposed in the main manuscript, and assuming $L_{\rm i} \geq L_0$, the longitudinal profile of the saturated flux can be expressed in a piece-wise manner in five regions:

$$q_{\rm sat} = \begin{cases} q^{\rm c}_{\rm sat} & \text{Region I, } x \leq 0, \quad [1a] \\ q^{\rm c}_{\rm sat} - \Delta q_{\rm sat} \dfrac{x}{L_0} & \text{Region II, } 0 \leq x \leq L_0, \quad [1b] \\ q^{\rm e}_{\rm sat} & \text{Region III, } L_0 \leq x \leq L_{\rm i}, \quad [1c] \\ q^{\rm e}_{\rm sat} + \Delta q_{\rm sat} \dfrac{x - L_{\rm i}}{L_0} & \text{Region IV, } L_{\rm i} \leq x \leq L_{\rm i} + L_0, \quad [1d] \\ q^{\rm c}_{\rm sat} & \text{Region V, } x \geq L_{\rm i} + L_0, \quad [1e] \end{cases}$$

where we have defined:

$$\Delta q_{\rm sat} = q^{\rm c}_{\rm sat} - q^{\rm e}_{\rm sat}. \quad [2]$$

With these expressions for $q_{\rm sat}$, we can integrate Eq. 2 of the main manuscript in these five regions, with the additional condition that q stays constant if erosion cannot take place on the consolidated bed. In region I, the flux is simply equal to the incoming flux: $q(x) = q_{\rm in}$. In region II, we obtain

$$q(x) = q_{\rm in}\, e^{-x/L_{\rm sat}} - \Delta q_{\rm sat} \frac{x}{L_0} + \left(q^{\rm c}_{\rm sat} + \Delta q_{\rm sat} \frac{L_{\rm sat}}{L_0} \right) \left(1 - e^{-x/L_{\rm sat}} \right). \quad [3]$$

At the upwind end, $q(0) = q_{\rm in}$. At the downwind end of region II, the flux is

$$q(L_0) = q^{\rm e}_{\rm sat} + \Delta q_{\rm sat} \frac{L_{\rm sat}}{L_0} \left[1 - \left(1 + \frac{L_0}{L_{\rm sat}} - \frac{q_{\rm in} - q^{\rm e}_{\rm sat}}{\Delta q_{\rm sat}} \frac{L_0}{L_{\rm sat}} \right) e^{-L_0/L_{\rm sat}} \right] \quad [4]$$

From these expressions, we can also deduce the upstream erosion zone of the patch, for which $q \leq q_{\rm sat}$ in this region II. Its length $L_{\rm e}$ is computed from the condition $q(L_{\rm e}) = q_{\rm sat}(L_{\rm e})$. Using the above expressions it solves into

$$\frac{L_{\rm e}}{L_{\rm sat}} = \ln \left(1 + \frac{q^{\rm c}_{\rm sat} - q_{\rm in}}{\Delta q_{\rm sat}} \frac{L_0}{L_{\rm sat}} \right). \quad [5]$$

Similarly, in region III, we have

$$q(x) = q(L_0)\, e^{-(x-L_0)/L_{\rm sat}} + q^{\rm e}_{\rm sat} \left(1 - e^{-(x-L_0)/L_{\rm sat}} \right). \quad [6]$$

At $x = L_{\rm i}$, using Eq. 4, we obtain

$$q(L_{\rm i}) = q^{\rm e}_{\rm sat} + \Delta q_{\rm sat} \frac{L_{\rm sat}}{L_0}\, e^{-(L_{\rm i}-L_0)/L_{\rm sat}} \left[1 - \left(1 + \frac{L_0}{L_{\rm sat}} - \frac{q_{\rm in} - q^{\rm e}_{\rm sat}}{\Delta q_{\rm sat}} \frac{L_0}{L_{\rm sat}} \right) e^{-L_0/L_{\rm sat}} \right] \quad [7]$$

Finally, in region IV, we have

$$q(x) = q(L_{\rm i})\, e^{-(x-L_{\rm i})/L_{\rm sat}} + \Delta q_{\rm sat} \frac{x - L_{\rm i}}{L_0} + \left(q^{\rm e}_{\rm sat} - \Delta q_{\rm sat} \frac{L_{\rm sat}}{L_0} \right) \left(1 - e^{-(x-L_{\rm i})/L_{\rm sat}} \right). \quad [8]$$

Importantly, this expression is only valid up to $x = L_{\rm d}$, defined as the location where q and $q_{\rm sat}$ are equal in region IV. A similar calculation to that of $L_{\rm e}$ gives

$$\frac{L_{\rm d}}{L_{\rm sat}} = \frac{L_{\rm i}}{L_{\rm sat}} + \ln \left(1 + \frac{q(L_{\rm i}) - q^{\rm e}_{\rm sat}}{\Delta q_{\rm sat}} \frac{L_0}{L_{\rm sat}} \right). \quad [9]$$

For the rest of region IV, and also in region V, the flux stays constant as no erosion of the consolidated is possible. This allows to define the output flux as

$$\begin{aligned} q_{\rm out} &= q(L_{\rm d}) = q_{\rm sat}(L_{\rm d}) \\ &= q^{\rm e}_{\rm sat} + \Delta q_{\rm sat} \frac{L_{\rm sat}}{L_0} \ln \left(1 + \frac{q(L_{\rm i}) - q^{\rm e}_{\rm sat}}{\Delta q_{\rm sat}} \frac{L_0}{L_{\rm sat}} \right), \end{aligned} \quad [10]$$

where we have substituted the expression (9) for $L_{\rm d}$ in that for the saturated flux (1d). For the estimate the mass balance of such a flat patch, we must compare $q_{\rm out}$ to $q_{\rm in}$. With the above expression and inserting the expression of $q(L_{\rm i})$ (7) in the above of $q_{\rm out}$, the condition $q_{\rm out} = q_{\rm in}$ gives the following limiting curve in the parametric plane $q_{\rm in}$ *vs* $L_{\rm i}$:

$$\frac{L_{\rm i}}{L_{\rm sat}} = \ln \frac{e^{L_0/L_{\rm sat}} - 1 - \frac{L_0}{L_{\rm sat}} + \frac{q_{\rm in} - q^{\rm e}_{\rm sat}}{\Delta q_{\rm sat}} \frac{L_0}{L_{\rm sat}}}{e^{\frac{q_{\rm in} - q^{\rm e}_{\rm sat}}{\Delta q_{\rm sat}} \frac{L_0}{L_{\rm sat}}} - 1}. \quad [11]$$

Short initial patch length. We now consider the case $L_\mathrm{i} \leq L_0$. The saturated flux profile then displays four regions only – the central one disappears – and we take:

$$q_\mathrm{sat} = \begin{cases} q^\mathrm{c}_\mathrm{sat} & \text{Region I, } x \leq 0, \quad [12a] \\ q^\mathrm{c}_\mathrm{sat} - \Delta q_\mathrm{sat} \dfrac{x}{L_0} & \text{Region II, } 0 \leq x \leq L_\mathrm{i}, \quad [12b] \\ q^\mathrm{c}_\mathrm{sat} + \Delta q_\mathrm{sat} \dfrac{x - 2L_\mathrm{i}}{L_0} & \text{Region III, } L_\mathrm{i} \leq x \leq 2L_\mathrm{i}, \quad [12c] \\ q^\mathrm{c}_\mathrm{sat} & \text{Region IV, } x \geq 2L_\mathrm{i}. \quad [12d] \end{cases}$$

In this case, the flux profile is the same in regions I and II as before, but its value at $x = L_\mathrm{i}$ is now

$$q(L_\mathrm{i}) = q^\mathrm{e}_\mathrm{sat} + \Delta q_\mathrm{sat} \frac{L_\mathrm{sat}}{L_0} \left[1 + \frac{L_0 - L_\mathrm{i}}{L_\mathrm{sat}} - \left(1 + \frac{L_0}{L_\mathrm{sat}} - \frac{q_\mathrm{in} - q^\mathrm{e}_\mathrm{sat}}{\Delta q_\mathrm{sat}} \frac{L_0}{L_\mathrm{sat}} \right) e^{-L_\mathrm{i}/L_\mathrm{sat}} \right]. \quad [13]$$

In new region III, the flux is

$$q(x) = q(L_\mathrm{i})\, e^{-(x-L_\mathrm{i})/L_\mathrm{sat}} + \Delta q_\mathrm{sat} \frac{x - L_\mathrm{i}}{L_0} + \left(q^\mathrm{c}_\mathrm{sat} - \Delta q_\mathrm{sat} \frac{L_\mathrm{i} + L_\mathrm{sat}}{L_0} \right) \left(1 - e^{-(x-L_\mathrm{i})/L_\mathrm{sat}} \right). \quad [14]$$

If $L_\mathrm{i} \leq L_\mathrm{e}$ (given by Eq. 5), then the whole patch is eroding. In this case, the output flux is $q_\mathrm{out} = q(L_\mathrm{i})$ (Eq. 13), and $q_\mathrm{out} \geq q_\mathrm{in}$.

Conversely, when $L_\mathrm{i} \geq L_\mathrm{e}$, the profile (14) is valid until q and q_sat are equal. The corresponding position L_d is

$$\frac{L_\mathrm{d}}{L_\mathrm{sat}} = \frac{L_\mathrm{i}}{L_\mathrm{sat}} + \ln \left[2 - \left(1 + \frac{L_0}{L_\mathrm{sat}} - \frac{q_\mathrm{in} - q^\mathrm{e}_\mathrm{sat}}{\Delta q_\mathrm{sat}} \frac{L_0}{L_\mathrm{sat}} \right) e^{-L_\mathrm{i}/L_\mathrm{sat}} \right]. \quad [15]$$

and the corresponding output flux is

$$q_\mathrm{out} = q^\mathrm{e}_\mathrm{sat} + \Delta q_\mathrm{sat} \frac{L_0 - L_\mathrm{i}}{L_0} + \Delta q_\mathrm{sat} \frac{L_\mathrm{sat}}{L_0} \ln \left[2 - \left(1 + \frac{L_0}{L_\mathrm{sat}} - \frac{q_\mathrm{in} - q^\mathrm{e}_\mathrm{sat}}{\Delta q_\mathrm{sat}} \frac{L_0}{L_\mathrm{sat}} \right) e^{-L_\mathrm{i}/L_\mathrm{sat}} \right]. \quad [16]$$

The condition $q_\mathrm{out} = q_\mathrm{in}$ now gives an implicit relation between q_in and L_i:

$$\frac{q_\mathrm{in} - q^\mathrm{e}_\mathrm{sat}}{\Delta q_\mathrm{sat}} \frac{L_0}{L_\mathrm{sat}} = \frac{L_0 - L_\mathrm{i}}{L_\mathrm{sat}} + \ln \left[2 - \left(1 + \frac{L_0}{L_\mathrm{sat}} - \frac{q_\mathrm{in} - q^\mathrm{e}_\mathrm{sat}}{\Delta q_\mathrm{sat}} \frac{L_0}{L_\mathrm{sat}} \right) e^{-L_\mathrm{i}/L_\mathrm{sat}} \right]. \quad [17]$$

All the flux profiles corresponding to the above different cases are displayed in Fig. S15

Supplementary figures

Fig. S1 complements Fig. 2 of main manuscript by showing the shear stress profile.
Fig. S2 complements Fig. 2 of main manuscript by showing the time evolution of the bedform's height, position and length.
Fig. S3 displays the model's behavior for three different L_0-values and $q_\mathrm{in} = 2q^\mathrm{e}_\mathrm{sat}$.
Fig. S4 displays the model's behavior for three different q_in-values and $L_0 = 2L_\mathrm{sat}$.
Fig. S5 displays the model's behavior for three different q_in-values and $L_0 = 0$.
Fig. S6 shows how the transition from the disappearing to the growing regimes is modified when the model's parameters are varied.
Fig. S7 shows the comparison of simulations near the transition from the disappearing to the growing regimes with the analytical solution considering flat beds.
Fig. S8 illustrates the effect of the two transport laws and the transition length L_0 on the steady propagative solutions.
Fig. S9 displays the model's behavior for low q_in-values.
Fig. S10 shows how the bedform evolution is affected by the initial length L_i.
Fig. S11 shows how the bedform evolution is affected by the initial height H_i.
Fig. S12 presents the comparison of the model with a growing and migrating bedform (see pink ellipse in Fig. 3 of main manuscript).
Fig. S13 presents the comparison of the model with a disappearing bedform.
Fig. S14 complements Fig. 1 of main manuscript by showing the distribution of the wind direction during the experiment on the 13th September 2022.
Fig. S15 shows the analytical flux profiles calculated in this SI appendix.
Fig. S16 displays an example of a spreading bedform.
Fig. S17 presents the detrending and smoothing of the TLS data.
Fig. S18 shows grain size distributions from various locations.

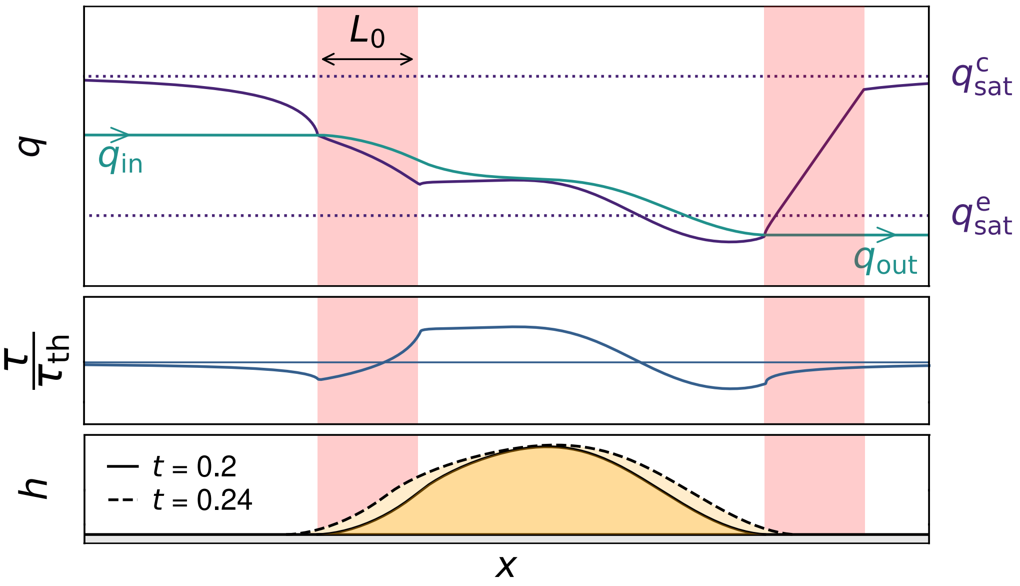

Fig. S1. **Shear stress profile for spreading bedforms.** Same as Fig. 2D of main manuscript, with the additional profile of the basal shear stress. Note the reduced τ both upwind and downwind of the bedform, due to the feedback of the topography on the flow (Eq. 3 of main manuscript). This also explains why $q(x) < q^{e}_{sat}$ downwind of the bedform.

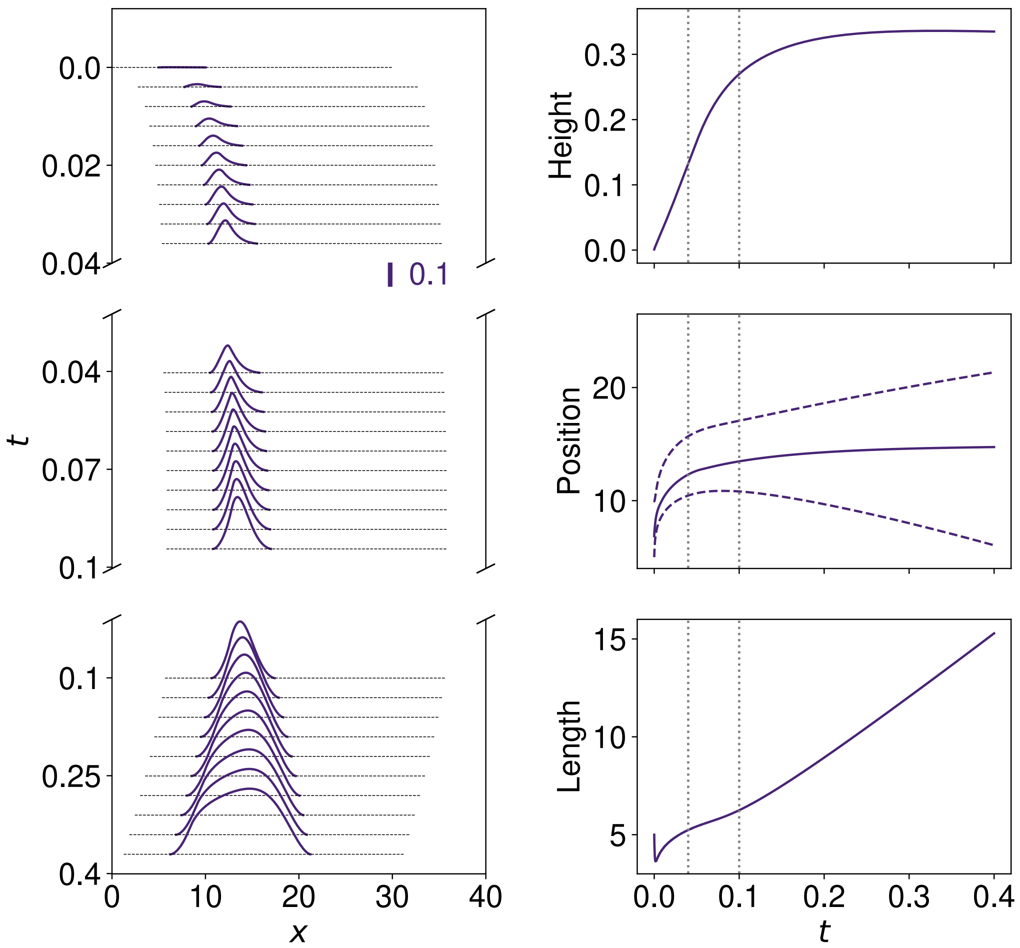

Fig. S2. Time evolution of a growing bedform. These graphs complement those of Fig. 2 of main manuscript, whose spatio-temporal diagram (panel E of that figure) is here reproduced on the left. On the right, we show the bedform height (top), the position of its crest (middle) as well as its upwind and downwind edges (represented by the dashed lines), and its length (bottom) as a function of time. Run for $\{L_0,\ q_{\rm in}/q_{\rm sat}^{\rm e}\} = \{2.0,\ 2.0\}$. The vertical dotted lines indicate the starting time of each sub-panel in the spatio-temporal diagram. Note that for $t > 0.1$, the spreading regime is reached : the height remains constant while the upwind and downwind edges of the bedform move apart, thus increasing the length.

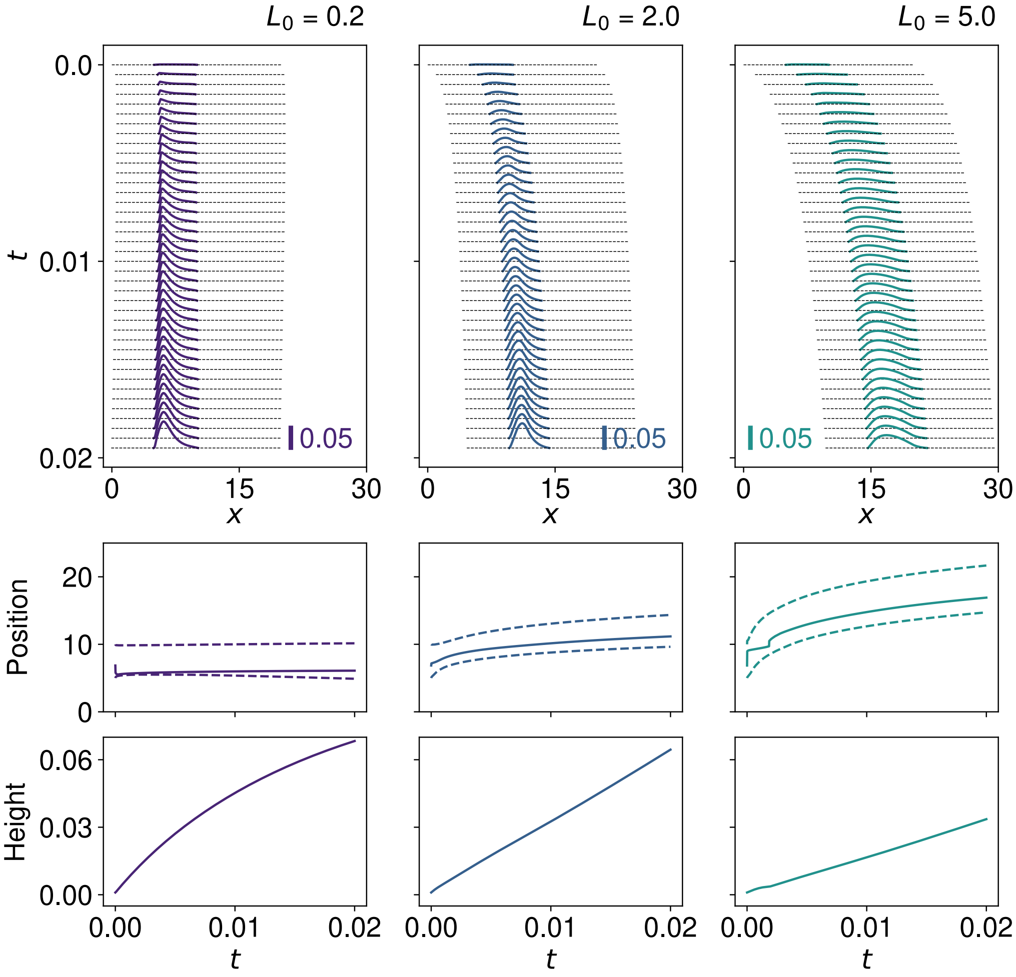

Fig. S3. **Influence of parameter L_0 on the evolution of the bedform.** Top : Spatio-temporal diagrams. Middle : time evolution of the bedform crest (plain line) and edge positions (dashed lines). Bottom : time evolution of the bedform height. Three values of L_0 are represented : 0.2 (left), 2 (middle) and 5 (right). All these runs are for $q_{\mathrm{in}} = 2\, q_{\mathrm{sat}}^{\mathrm{e}}$. For a given q_{in}, the increase in L_0 leads to greater erosion at the upwind edge (see also Eq. 5 of SI text) and therefore reinforces the migration rate of the bedform. Furthermore, as L_0 increases, the bedform becomes less abrupt and its height decreases, as the sand is more evenly distributed over a greater distance.

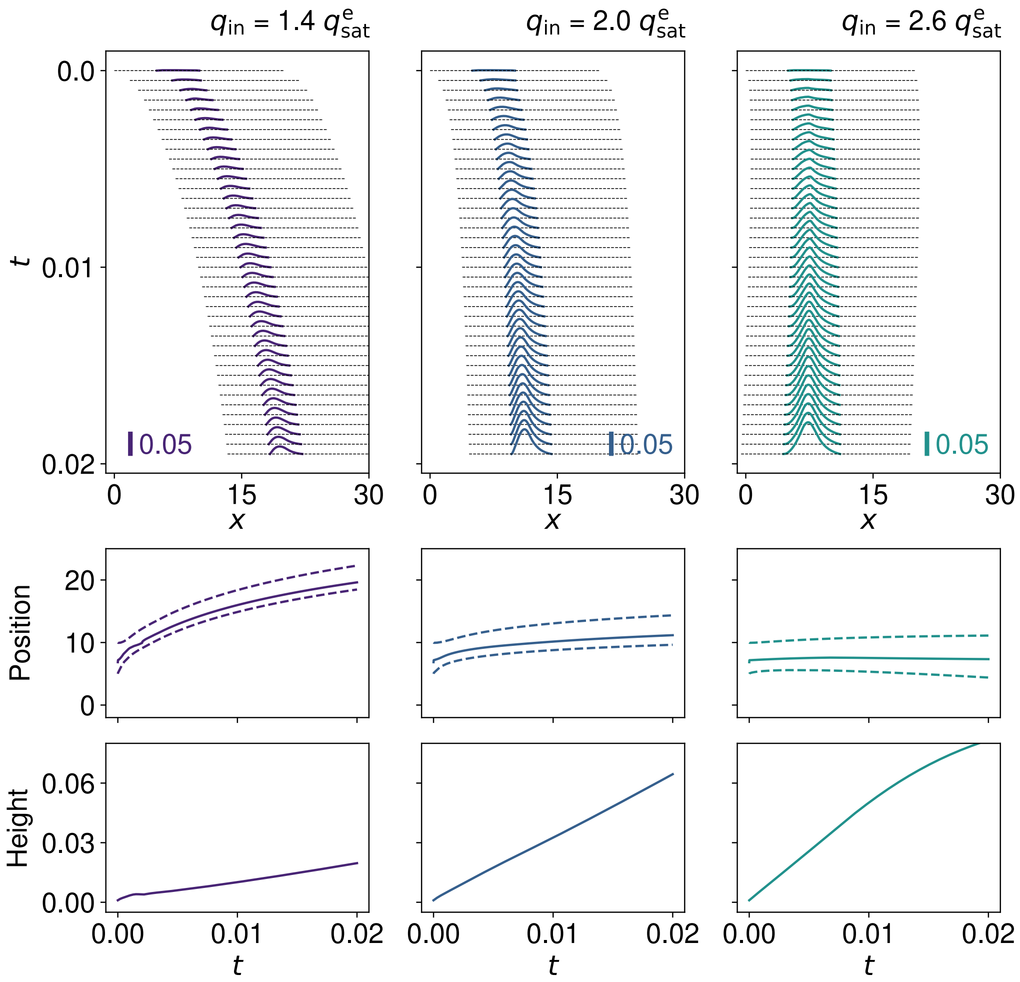

Fig. S4. Influence of parameter q_{in} on the evolution of the bedform. Top : Spatio-temporal diagrams. Middle : Time evolution of the bedform crest (plain line) and edge position (dashed lines). Bottom : time evolution of the bedform height. Three values of q_{in} are represented : $1.4\, q_{\mathrm{sat}}^{\mathrm{e}}$ (left), $2\, q_{\mathrm{sat}}^{\mathrm{e}}$ (middle) and $2.6\, q_{\mathrm{sat}}^{\mathrm{e}}$ (right). All these runs are for $L_0 = 2$. For a given L_0, the increase in q_{in} leads to less erosion at the upwind edge (see also Eq. 5 of SI text) and therefore reduces the migration rate of the bedform. In addition, as q_{in} increases, the bedform height becomes greater, as more sand is deposited.

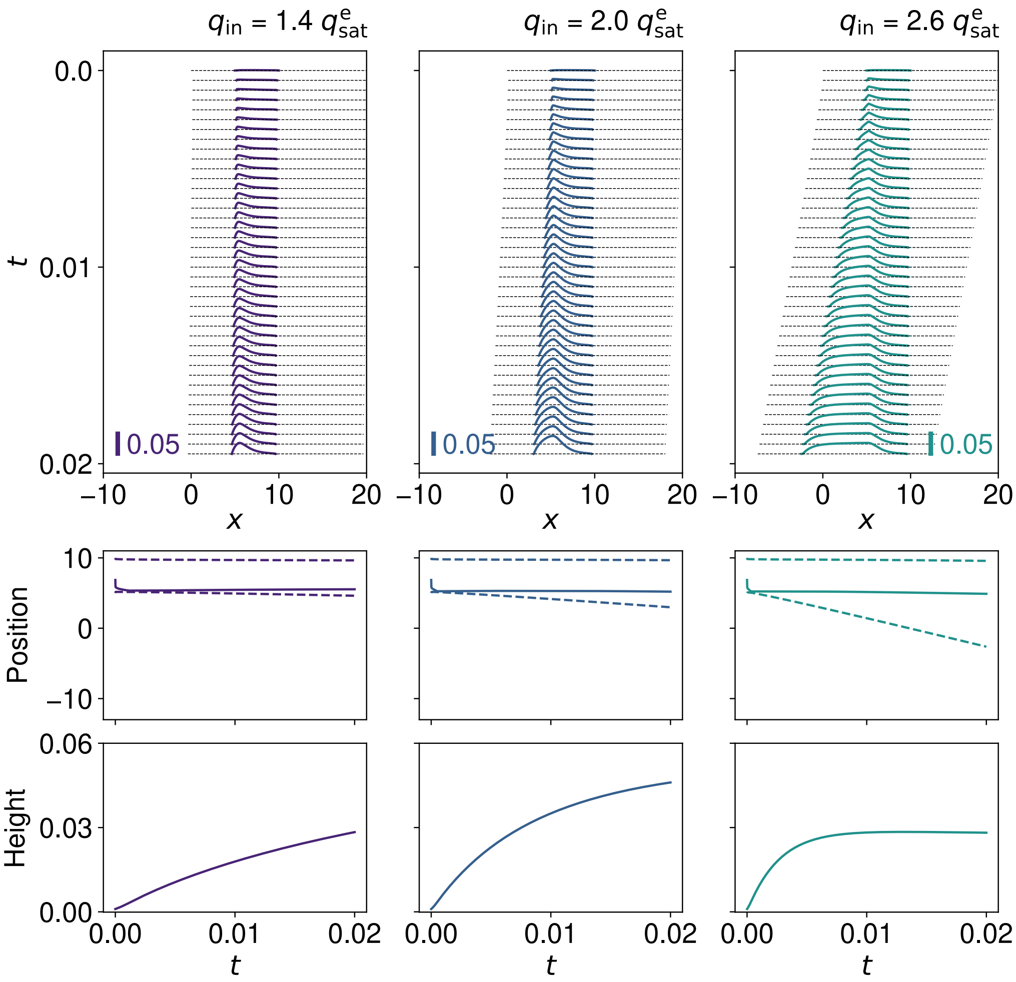

Fig. S5. Spreading dynamics of the bedform for $L_0 = 0$. Same as Fig. S4, but for the special case $L_0 = 0$. In this case, there is only deposition at the upwind edge. Increasing q_{in} leads to more deposition and therefore greater spreading.

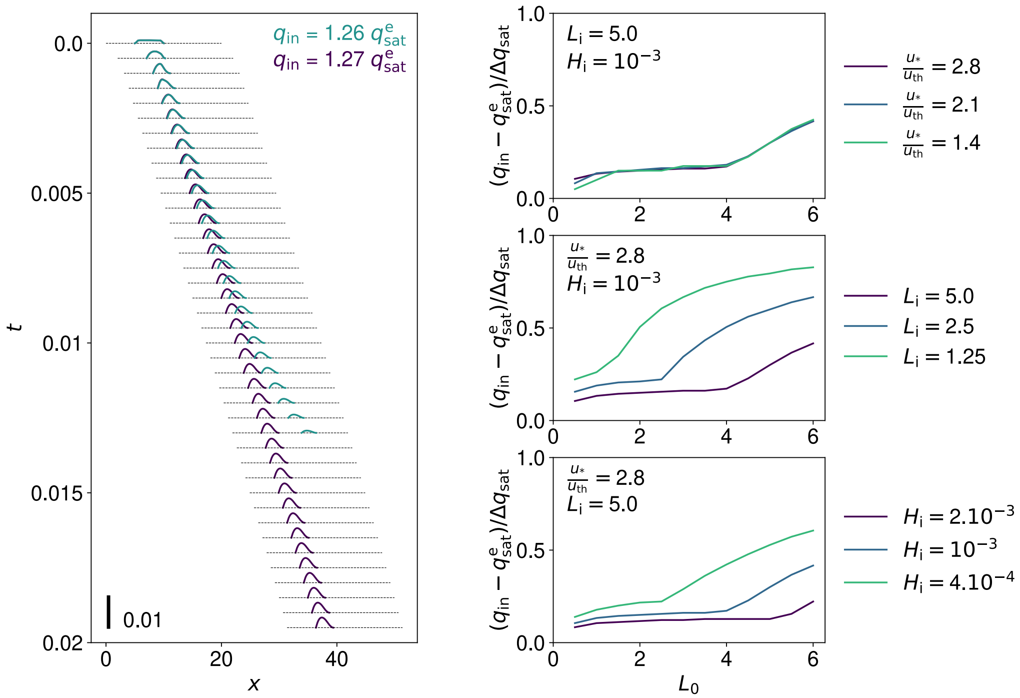

Fig. S6. Influence of the initial conditions and wind speed on the limit between the growing and disappearing regimes. Left: Spatio-temporal diagram showing the evolution of the bedform just above and below the growth limit shown as a white line in Fig. 3 in main manuscript. These runs are for $L_0 = 2$, $u_*/u_{\rm th} = 2.8$, $L_{\rm i} = 5$ and $H_{\rm i} = 10^{-3}$. Right: Influence of wind speed, initial length and height (see values in legends) on this growth limit. Top: $L_{\rm i} = 5$ and $H_{\rm i} = 10^{-3}$. Middle: $u_*/u_{\rm th} = 2.8$ and $H_{\rm i} = 10^{-3}$. Bottom: $u_*/u_{\rm th} = 2.8$ and $L_{\rm i} = 5$. As shown in the top right panel, any dependence on wind velocity is accounted for once expressed in fluxes. The curves in the middle and bottom right panels show a first flat regime, independent of L_0, and then an increase of the input flux necessary to get the bedform growing. We find that the L_0-value of the transition depends on the initial mass of the bedform: it is larger for a larger mass. In other words, the dynamics starts to be sensitive to L_0 when the initial bedform mass is small, which requires a significantly larger input flux to grow.

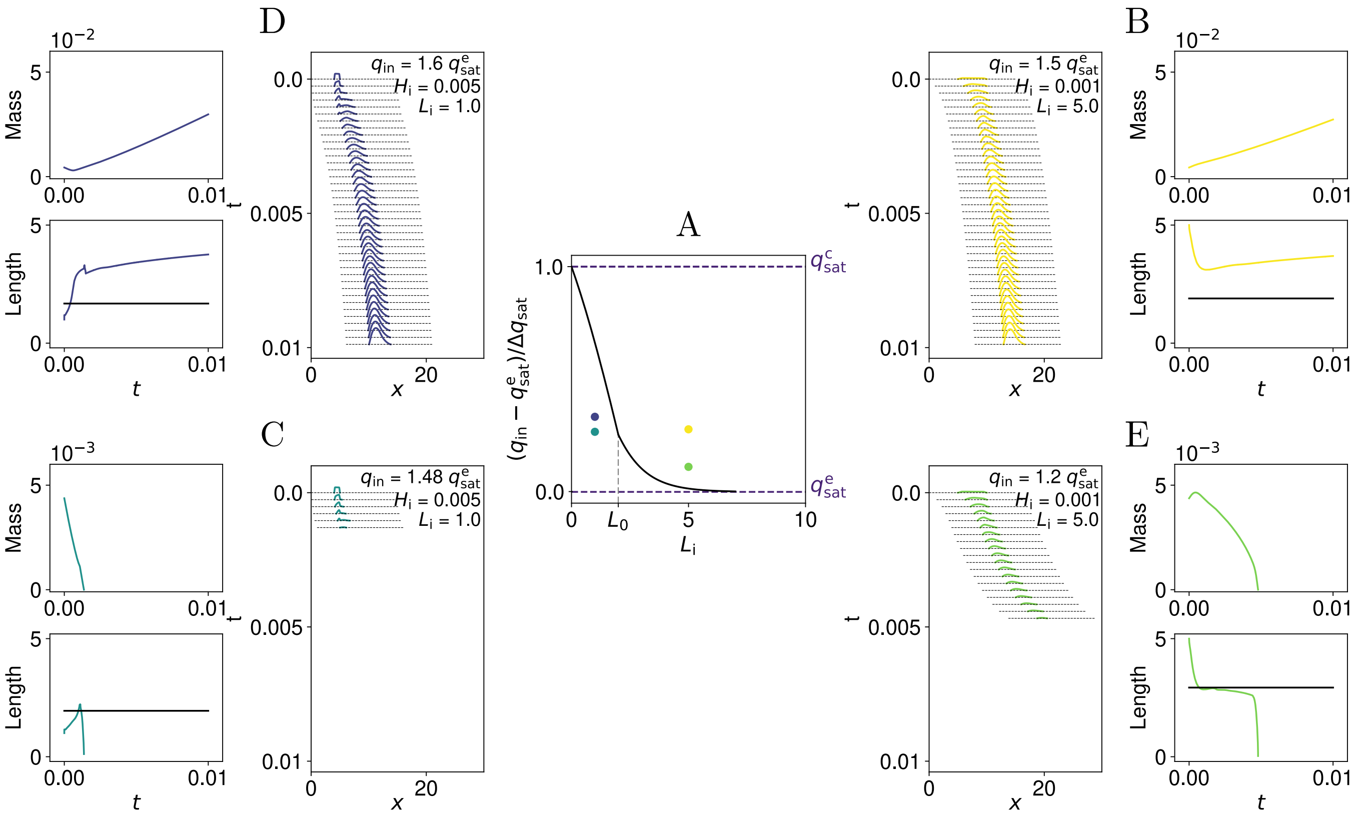

Fig. S7. Comparison of simulations near the limit between the growing and disappearing regimes with the analytical solutions for flat case. Central panel $\mathbf{A}$: Parametric plane showing patch initial length L_i *versus* input flux (normalized as $(q_{\rm in} - q_{\rm sat}^{\rm e})/\Delta q_{\rm sat}$, where $\Delta q_{\rm sat} = q_{\rm sat}^{\rm c} - q_{\rm sat}^{\rm e}$) to show four different cases ($\mathbf{B}$-$\mathbf{E}$, see corresponding colors and parameter values in legend). The black line represents Eqs. 11, and 17 of SI text associated with a mass-balanced flat patch ($q_{\rm in} = q_{\rm out}$). On the left of this curve, mass balance is negative ($q_{\rm in} < q_{\rm out}$), whereas it is positive on the right ($q_{\rm in} > q_{\rm out}$). In practice, the bedforms have a finite thickness, and this critical line is an approximation of a wider transition zone. Lateral panels ($\mathbf{B}$-$\mathbf{E}$): spatio-temporal diagrams of these four cases, and time evolution of the bedform mass and length. In the panels for the length, the solid black line also represents the analytical limit $q_{\rm in} = q_{\rm out}$. All runs are for $L_0 = 2.0$. A typical growing and migrating bedform is shown in case $\mathbf{B}$: it starts on the right of the mass-balanced curve, and gains mass with time. Meanwhile, its length, sufficiently larger than L_0, reduces as expected from the analytics (see also Fig. S15$\mathbf{A}$), and then gently increases, always staying well above the critical size (black line). A typical disappearing bedform is shown in case $\mathbf{C}$: it starts on the left of the mass-balanced curve, and looses mass with time. Its small initial length first increases, as expected from the analytics (see also Fig. S15$\mathbf{D}$), but its initial mass and the input flux were insufficient to make it cross the critical line significantly. The bedform then eventually dies. By contrast, case $\mathbf{D}$ illustrates the example where the input flux was larger, and sufficient to allow the bedform to increase its length well over the critical size. As a result, even if its mass was initially decreasing, it eventually accumulates sand and grows. Finally, in case $\mathbf{E}$, the bedform initially gains mass while reducing its length as in $\mathbf{B}$, but the input flux was not sufficient to prevent it to cross the critical size.

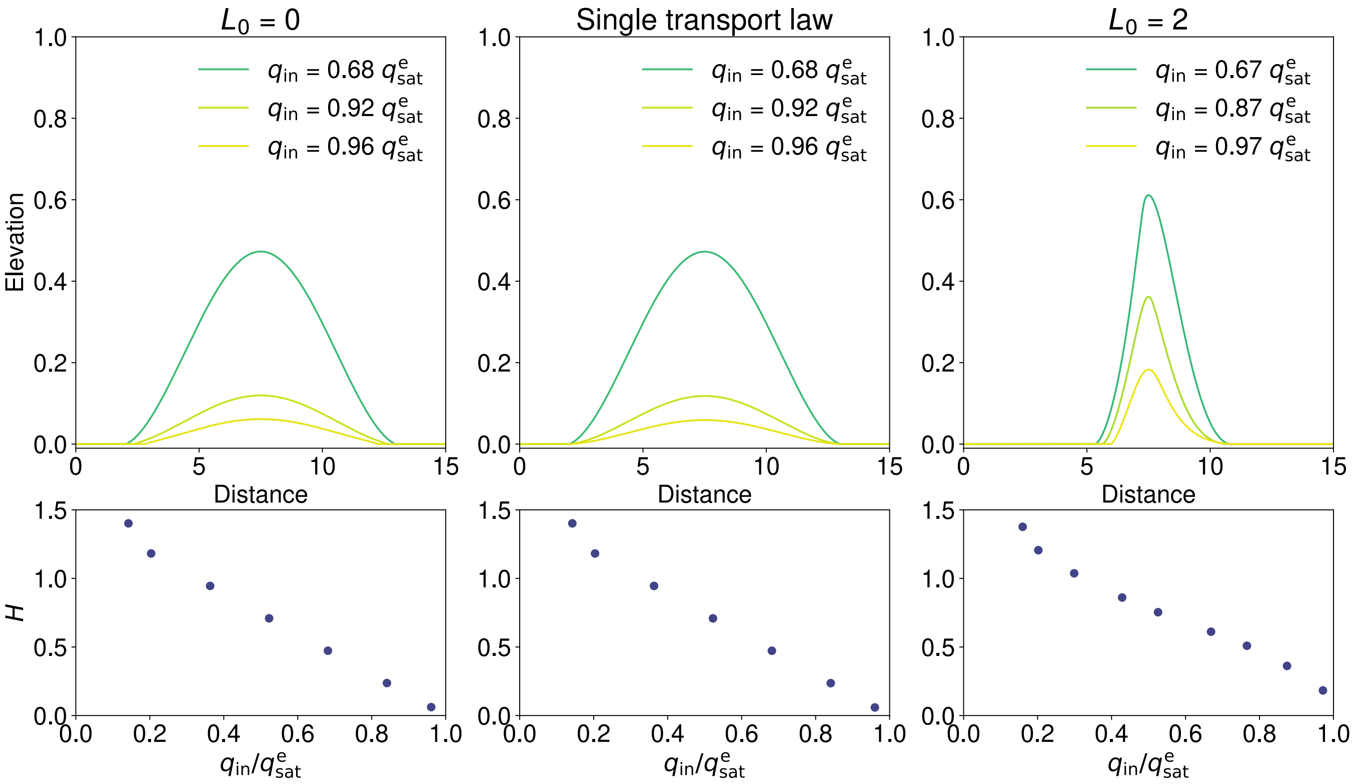

Fig. S8. Steady propagative solutions at low incoming flux Stationary states (i.e. with the condition $q_{\rm in} = q_{\rm out}$) of the equations exist at low input flux: $q_{\rm in} < q^{\rm e}_{\rm sat}$. Top: corresponding elevation profiles $h(x)$ at different values of $q_{\rm in}$ (see legends) in the case of $L_0 = 0$ (left), for a single transport law (Eq. 4 of main manuscript) independent on the bed nature (middle), and for $L_0 = 2$ (right). Note the asymmetric profiles and reduced length in this later case, in comparison to the first two. Bottom: Decreasing crest height H of these profiles as a function of $q_{\rm in}/q^{\rm e}_{\rm sat}$. The profiles in the left and middle panels are identical as, for these low flux input conditions, the possibility of having a flux $q(x)$ larger than $q^{\rm e}_{\rm sat}$ never occurs when $L_0 = 0$.

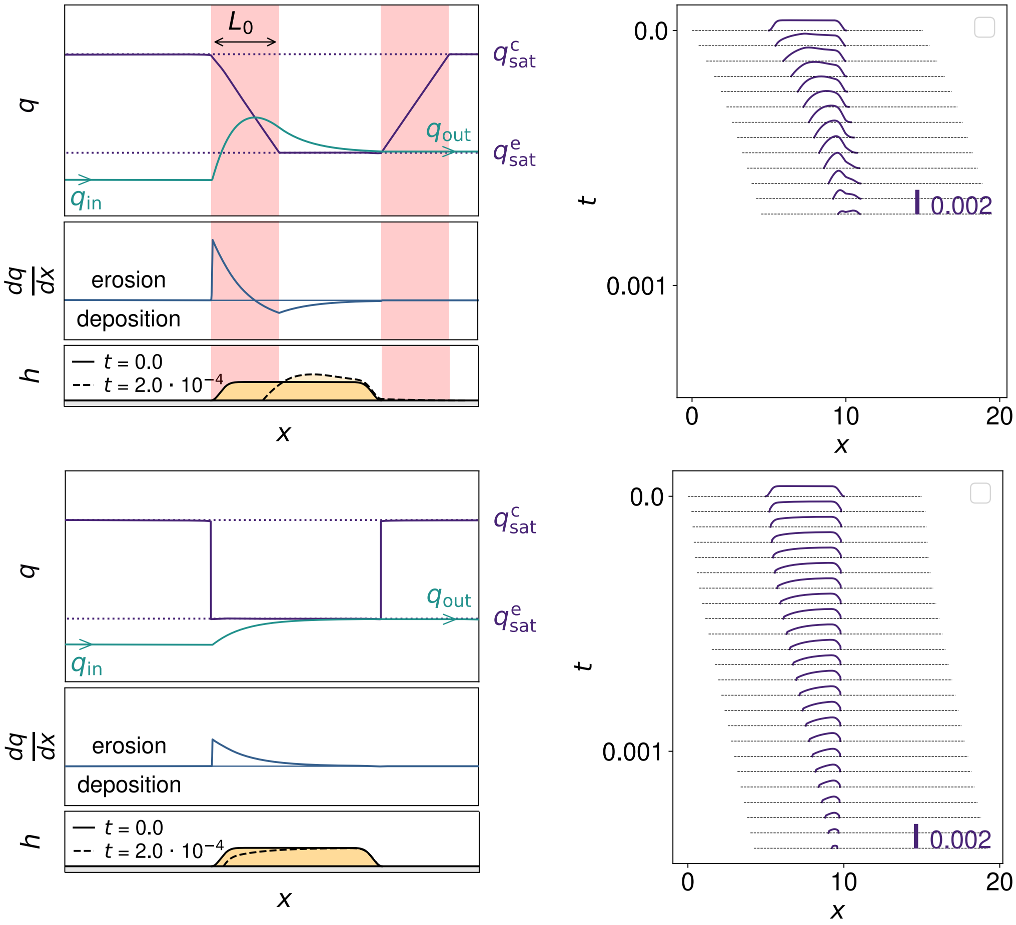

Fig. S9. **Dynamics of the bedform at low incoming sand flux**. Schematic profiles and spatio-temporal diagrams as in Fig. 2 of main manuscript for $L_0 = 2$ (top) and $L_0 = 0$ (bottom). In both cases, the bedform shrinks, as $q_{\rm out} \simeq q_{\rm sat}^{\rm e}$ is larger than $q_{\rm in}$. Their dynamics is not very different, except for a slightly enhanced upwind erosion for a finite L_0.

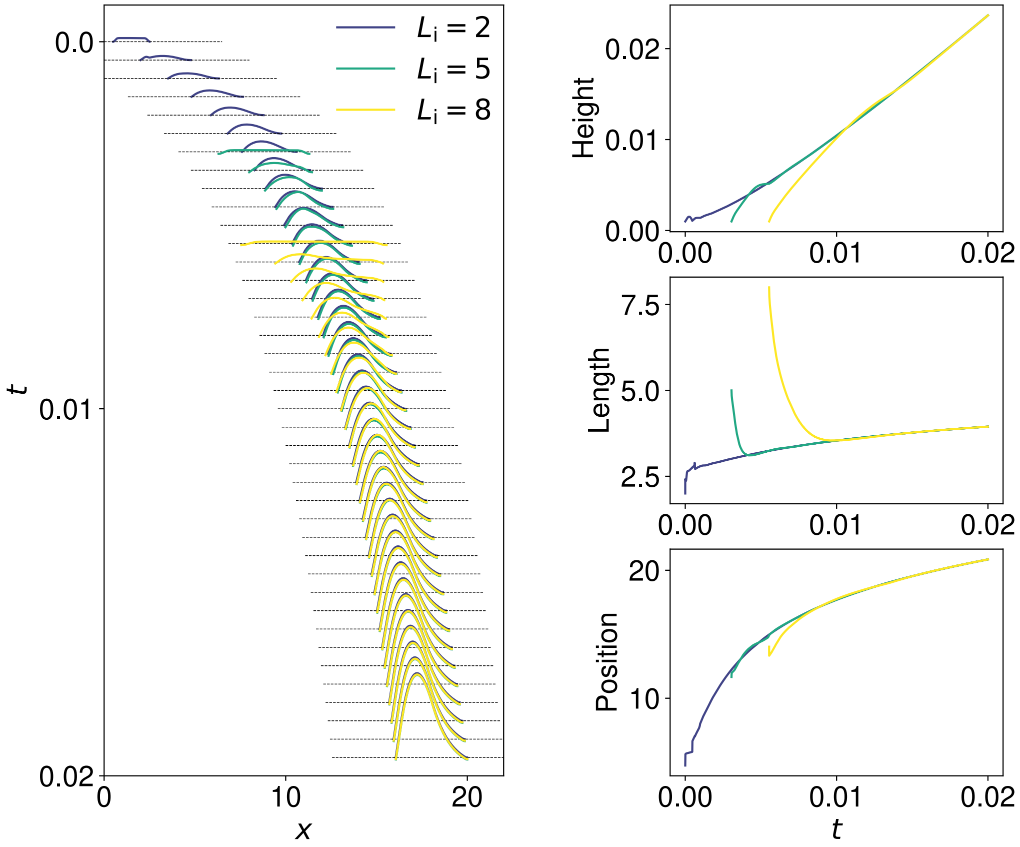

Fig. S10. Influence of the initial length L_i on the evolution of the bedform. Spatio-temporal diagram (left) and time evolution of the bedform height (top), length (middle) and position (bottom) for three values of $L_\mathrm{i} = \{2, 5, 8\}$. All these runs are for $L_0 = 2$ and $q_\mathrm{in} = 1.5\, q^\mathrm{e}_\mathrm{sat}$. Despite significant differences in shape over short times, the morphodynamics of the bedform eventually becomes independent of L_i.

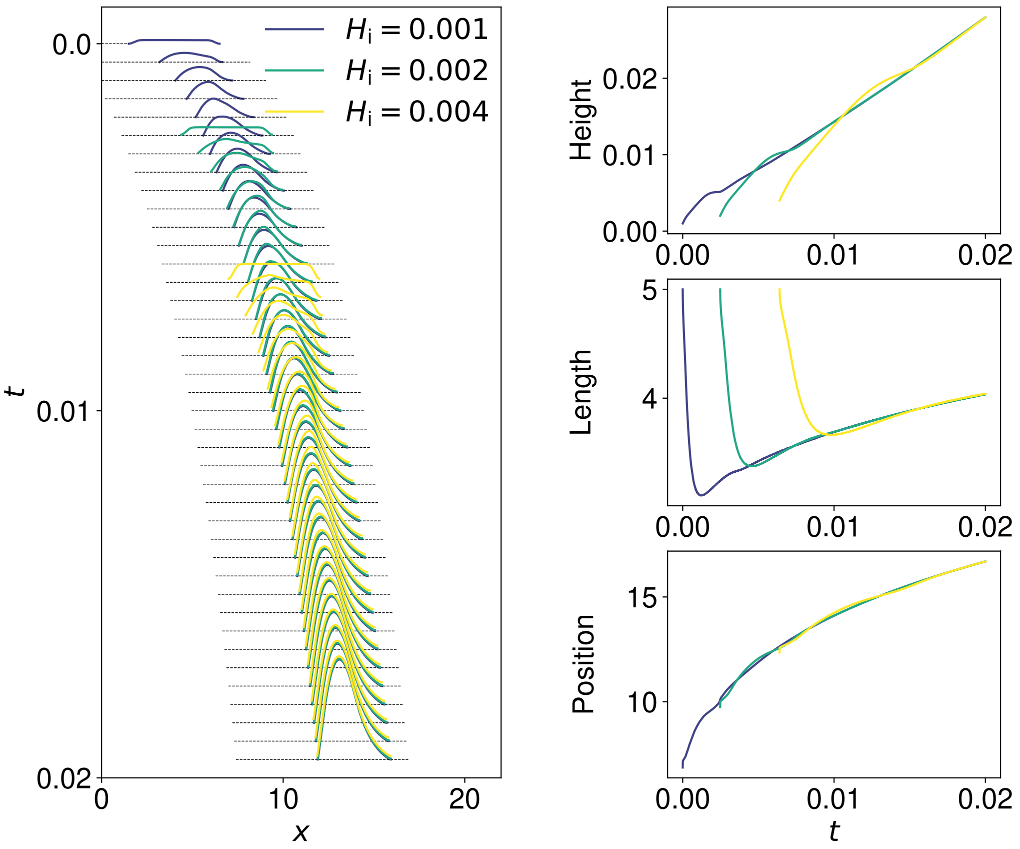

Fig. S11. **Influence of the initial height H_i on the evolution of the bedform.** Spatio-temporal diagram (left) and time evolution of the bedform height (top), length (middle) and position (bottom) for three values of $H_\mathrm{i} = \{0.001, 0.002, 0.004\}$. All these runs are for $L_0 = 2$, $q_\mathrm{in} = 1.5\, q_\mathrm{sat}^\mathrm{e}$. Despite significant differences in shape over short times, the morphodynamics of the bedform eventually becomes independent of H_i.

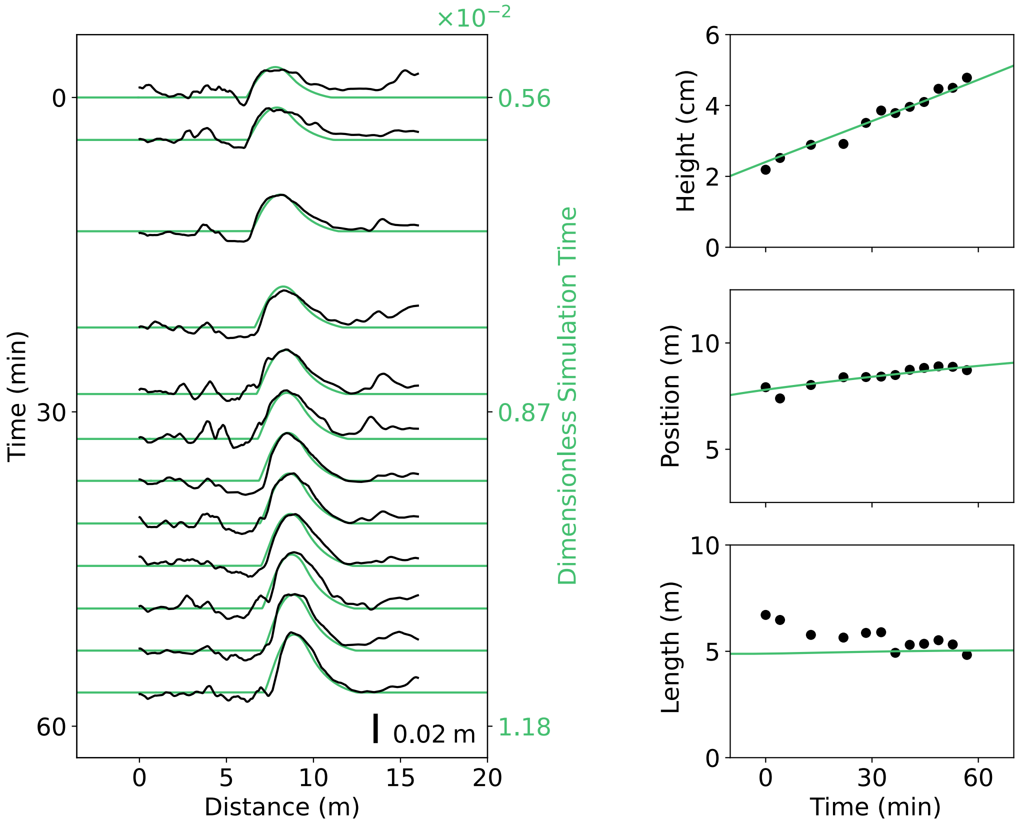

Fig. S12. **Model adjustment to field data showing the growth and migration of a meter-scale bedform.** Same as Fig. 4 of main manuscript for another bedform nearby (same constant wind). Time origin is 10:04 am on the 13th September 2022. The green solid lines are from the model with parameter values $\{L_0/L_{\mathrm{sat}},\ q_{\mathrm{in}}/q^{\mathrm{e}}_{\mathrm{sat}}\} = \{2.0,\ 2.0\}$. The ensemble of parameter values giving a reasonable fit of the data are displayed by the pink ellipse in Fig. 3 of the main manuscript. Right axis in green: dimensionless time in the simulation.

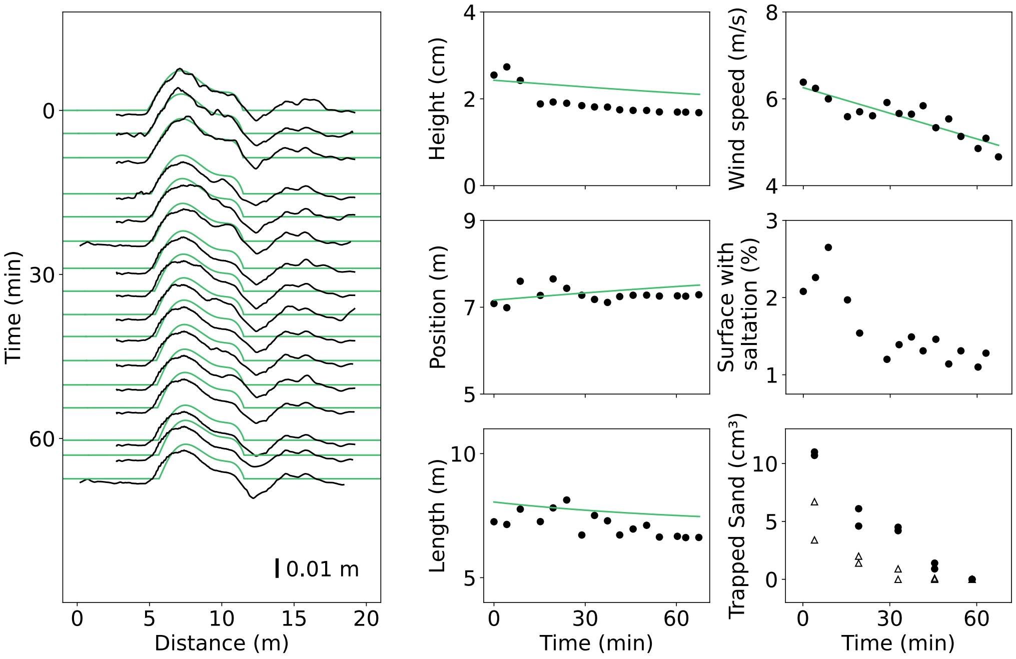

Fig. S13. **Model adjustment to field data showing a disappearing meter-scale bedform.** Same as Fig. 4 of main manuscript and Fig. S12, but here for the case of a decreasing wind. Time origin is 10:58 am on the $12^{\rm th}$ September 2023. The green solid lines are from the model with best fit parameter values $\{L_0/L_{\rm sat},\, q_{\rm in}/q^{\rm e}_{\rm sat}\} = \{0.0,\, 0.5\}$. Note that as $q^{\rm e}_{\rm sat}$ depends on the wind shear velocity (Eq. 4 of main manuscript), the input flux $q_{\rm in}$ decreases in proportion. A finer adjustment would consist of changing $q_{\rm in}$ at a different rate than $q^{\rm e}_{\rm sat}$. Besides, panels in the right column display time variations of wind speed measured at 0.24 m height, the percentage of the surface where saltation was detected during TLS scan, and sand captured in traps downwind (●) and sideways (△) of the bedform. This provides evidence of sediment transport during this period, decreasing and almost vanishing after one hour. Comparing data from the two sand traps, there is a larger flux out of the bedform than on its side, which gives a proxy for the flux upwind of the bedform. This is consistent with a shrinking dynamics.

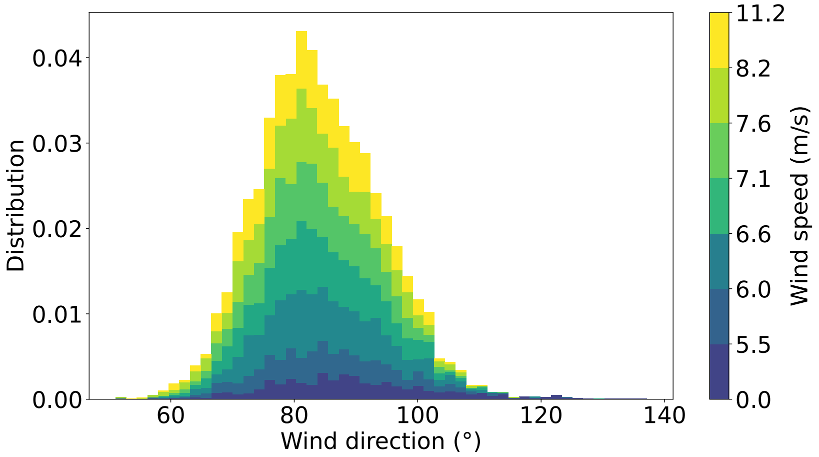

Fig. S14. Wind orientation distribution. Wind speed and orientation corresponding to the measurements on the 13th September 2022 (Fig. 1B,C). Data cumulated over the whole period of profile acquisition. Wind orientation is given in degrees anticlockwise from North. This peaked distribution corresponds to a unimodal wind regime with a mean orientation of $84°$ (easterly wind) with an angular dispersion of $7°$.

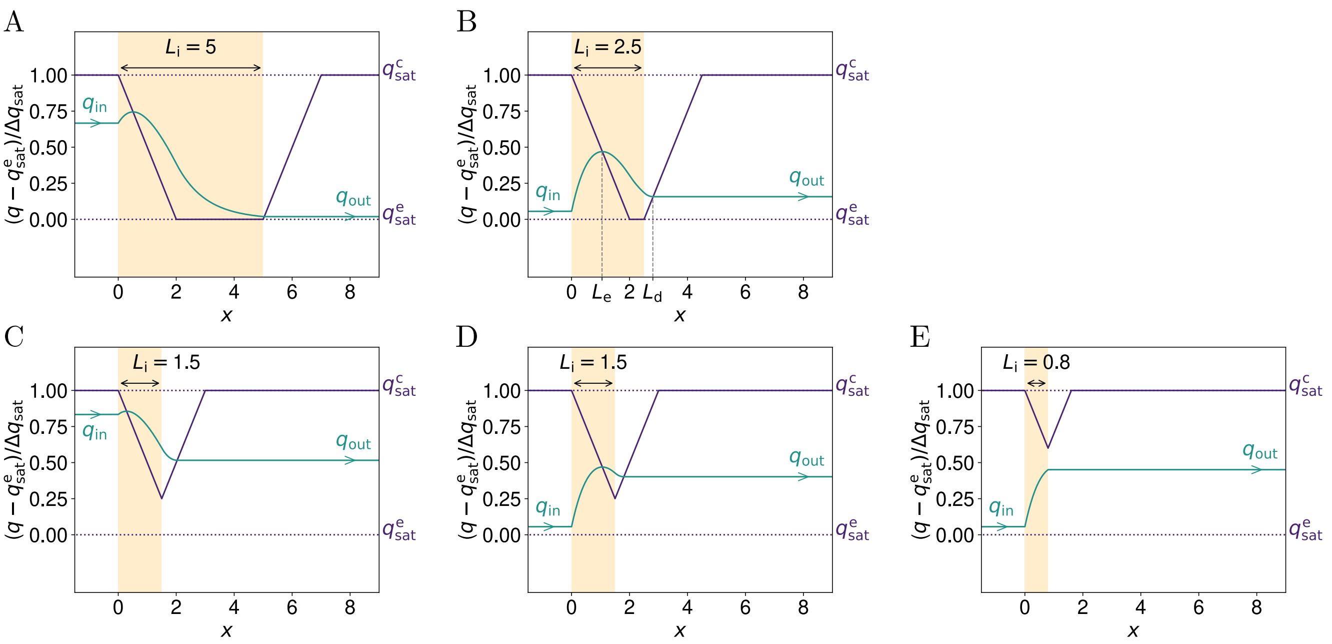

Fig. S15. Sand flux profiles from analytical calculations associated with perfectly flat beds. Profiles computed from expressions in SI text, with $L_0 = 2$, $q_\mathrm{sat}^\mathrm{c} = 2.8 q_\mathrm{sat}^\mathrm{e}$, and various patch lengths L_i (see values in legend) and input fluxes: (**A**) $q_\mathrm{in} = 2.2 q_\mathrm{sat}^\mathrm{e}$ and long initial patch length $L_\mathrm{i} > L_0$. In this case, $q_\mathrm{in} > q_\mathrm{out}$, as the output flux is close to $q_\mathrm{sat}^\mathrm{e}$. The zone eroded at the upwind edge (length L_e) is larger than the deposition zone downwind of the bedform (length $L_\mathrm{d} - L_\mathrm{i}$). Such a patch will gain mass as well as reduce in length. (**B**) $q_\mathrm{in} = 1.1 q_\mathrm{sat}^\mathrm{e}$ and long initial patch length $L_\mathrm{i} > L_0$. Here $q_\mathrm{in} < q_\mathrm{out}$. Such a patch will both lose mass and reduce in length. Dashed lines : display of the lengths L_e and L_d. (**C**) $q_\mathrm{in} = 2.5 q_\mathrm{sat}^\mathrm{e}$ and short initial patch length $L_\mathrm{i} < L_0$ with $L_\mathrm{i} > L_\mathrm{e}$. Here the patch will gain mass as $q_\mathrm{in} > q_\mathrm{out}$, but in contrast to case **A**, the erosion zone upwind is smaller than the deposition zone downwind, and it will consequently increase in length. (**D**) $q_\mathrm{in} = 1.1 q_\mathrm{sat}^\mathrm{e}$ and short initial patch length $L_\mathrm{i} < L_0$ with $L_\mathrm{i} > L_\mathrm{e}$. This case behaves similarly to **B**. (**E**) $q_\mathrm{in} = 1.1 q_\mathrm{sat}^\mathrm{e}$ and short initial patch length $L_\mathrm{i} < L_0$ with $L_\mathrm{i} = L_\mathrm{e}$. The whole patch is in the erosion zone, and will disappear.

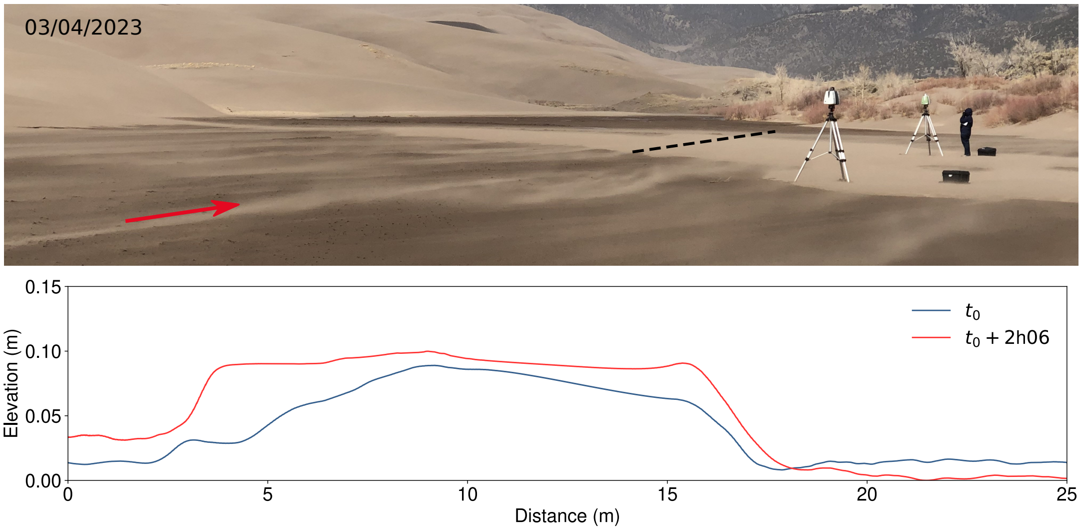

Fig. S16. $\mathbf{Spreading\ dynamics}$. Top : Photo of a spreading bedform at Great Sand Dunes National Park (USA) on the 3$^{\rm rd}$ April 2023. Here the consolidated surface is moist sand. The averaged wind speed $u = 9.33$ m/s was measured 220 m away from the bedform at $z = 0.24$ m using a 3D Sonic anemometer. Red arrow indicates wind direction. Black dashed line shows the approximate position of displayed transects. Bottom : Elevation profiles of the bedform measured over 2 hours and 06 minutes, starting at t_0 =12:18. Sand accumulates at both upwind and downwind toes of the bedform, showing a spreading dynamics. Note the relatively large scale of this bedform, in comparison to those in the growing and migrating regime observed in Namibia (Fig. 4).

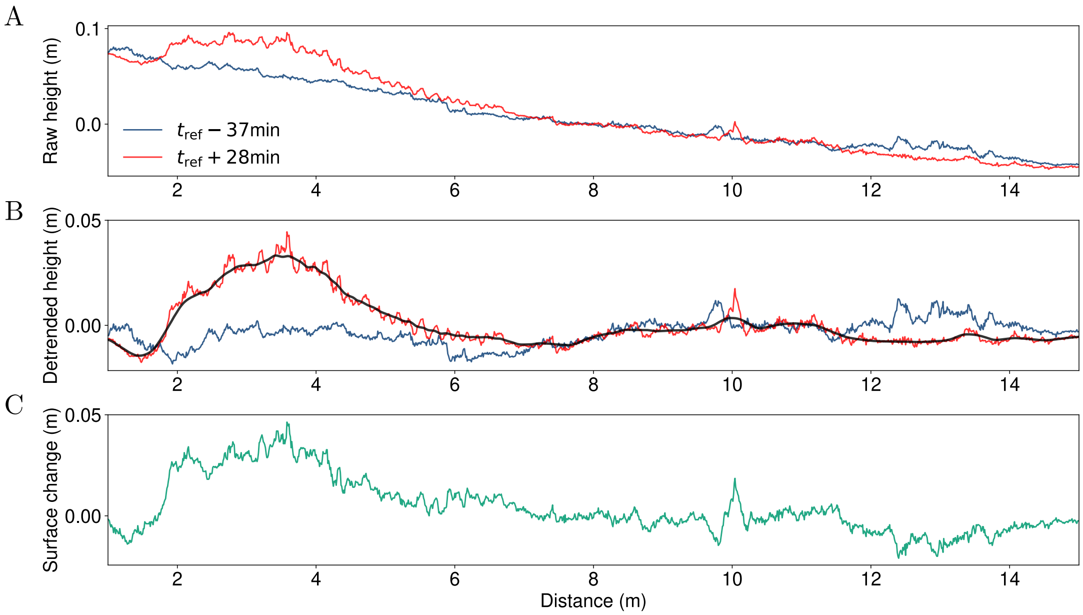

Fig. S17. Detrending and smoothing data processes. Illustration of data processing to obtain elevation profiles of the bedform displayed in Fig. 4 of main manuscript. t_{ref} corresponds to 9:40 am on the 13th September 2022. (**A**) Elevation profiles as obtained by the TLS raw signal once filtered to remove saltation and gridded at 1 cm horizontal resolution. Blue line: initial surface before bedform emergence (09:03 am). Red line: Bedform at time 10:08 am. (**B**) Detrending: the underlying topography is subtracted as explained in the section describing the field measurements method in the main manuscript. Smoothing: the black line is the result of a 45 cm mean moving window filter on the red profile in order to remove the sand ripples. This detrended and smooth profile is displayed in Fig. 4 (8th profile) and has been fitted by the model. (**C**) Surface difference where the blue profile is subtracted from the red one, in order to emphasize erosion and deposition with respect to the consolidated bed.

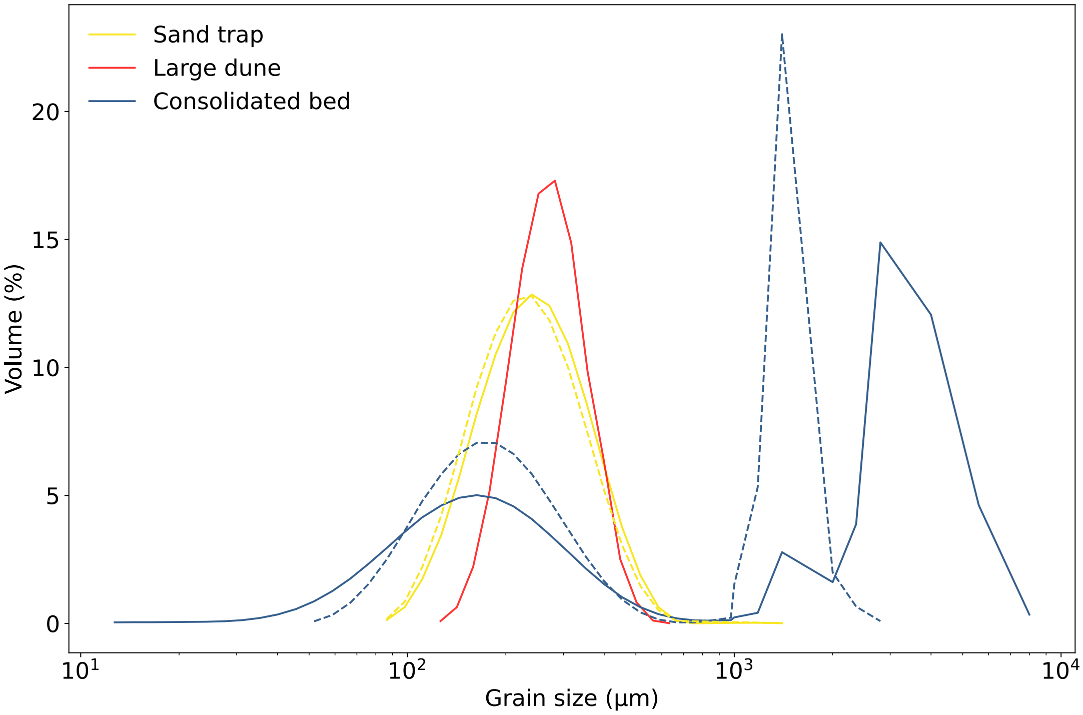

Fig. S18. **Grain size distributions**. Size distributions of saltating particles collected in sand traps (yellow), on a nearby large dune located at 15°01'35.14"E, 23°34'35.48"S (red), and on the consolidated bed in the interdune area (blue). Sand traps: one was located next to a bedform (that displayed in Fig. S13) but on the consolidated bed; the other one was deployed on a nearby flat sand bed. The close correspondence of the two yellow curves demonstrates that saltating grains are the same on both bed types. Large dune: the red distribution is similar to the yellow ones, which suggests that the transported grains come from the neighboring dune. Consolidated bed: the distributions are clearly bimodal, with a peak at small size similar to the sand bedforms, and peaks associated with millimeter-size gravels. These large particles typically represent $\simeq 40\%$ of the bed material. The two blue curves correspond to two similar places between the bedforms, but show a spatial variation in gravel size.
